# Some Intracranial Vascular Lesions

**Published:** 1909-08-14

**Authors:** W. Langdon Brown

**Affiliations:** Physician to the Metropolitan Hospital; Medical Registrar and Demonstrator of Physiology, St. Bartholomew's Hospital


### Hospital Clinics.

### SOME INTRACRANIAL VASCULAR LESIONS.

By W. LANGDON BROWN, M.A., M.D. Cantab, F.R.C.P.; Physician to the Metropolitan Hospital; Medical Registrar and Demonstrator of Physiology, St. Bartholomew's Hospital.

(Delivered before the Hunterian Society, November 1908.)

It has been frequently observed that remarkable cases, like misfortunes, rarely come singly. Recently I saw within three weeks the following series of intracranial vascular lesions, presenting unusual features. My reason for putting them on record is to call attention to the valuable aid to diagnosis which was afforded by manometric observation of the blood-pressure. It will be noted that, when the other signs appeared to point to a different conclusion from that arrived at by the manometer, events proved that the manometer was right.

### Ruptured Intracranial Aneurysm.

This is not a very common cause of cerebral hæmorrhage, yet within a week I had the opportunity of seeing two cases. In his first 800 autopsies at Montreal, Osler met with twelve cases of aneurysm of the cerebral arteries, three of which originated in the anterior communicating. This is a considerably larger proportion than in Newton Pitt's series of nineteen cases of aneurysm of the cerebral arteries out of 9,000 post-mortems.

The first case was under the care of my colleague, Dr. Leonard Williams, at the Metropolitan Hospital. She was a woman of 65, who had been attending the out-patient department for several months, with the usual symptoms of high tension. Her blood-pressure was 220 mm. Under treatment her symptoms gradually abated, and the manometer registered 160 mm. only. One day she brought a friend to the hospital who proved to be suffering from diphtheria. This agitated her very much, and she suddenly fell down and became completely unconscious. Her blood-pressure was now found to have risen to about 245 mm. This sudden rise pointed most strongly to cerebral hæmorrhage, as, indeed, did the other signs. A few hours later she died, without regaining consciousness. Post-mortem a ruptured aneurysm about the size of a pea was found on the anterior communicating artery, and a large quantity of blood extravasated all over the base of the brain. The kidneys were granular, though the heart was natural in size.

The same week I made a post-mortem examination on a man who had been admitted to St. Bartholomew's Hospital under Dr. Norman Moore. He was 41 years old, and had been found unconscious in the street by the police. Soon after he was brought in, violent left-sided convulsions occurred, and he died in a few hours. An exactly similar state of affairs to the last case was found; but in addition to the blood extravasated at the base of the brain, blood had tracked up the right Sylvian fissure, and by its pressure on the motor area had produced the convulsions. The heart was hypertrophied and the kidneys were granular.

As a contrast to these cases I may quote one which I saw under the care of my colleague, Dr. Haig, at the Metropolitan Hospital.

### Cerebral Softening Following Cerebral Embolism.

A woman of 40 was admitted for a sudden left hemiplegia. She did not lose consciousness at once, but after a few hours she passed into ingravescent coma. There were signs of mitral stenosis, and the blood-pressure was only 110 mm. The coma became so deep that I wondered whether a secondary hæmorrhage could have occurred from the softened artery subsequently to the obstruction. But in view of the fact that the pressure remained at 110, this seemed to be impossible. She lived for several days without regaining consciousness. At the post-mortem an area of red softening was found in the right lenticular nucleus, involving its posterior three-quarters and the adjacent part of the internal capsule. In the heart the cusps of the mitral valve were fused together, so that the orifice only admitted one finger. There were two small anæmic infarcts in the kidney. In my experience it is unusual for embolism to lead to such profound coma; it is interesting to note that the blood-pressure pointed emphatically to the conclusion that hæmorrhage was not responsible for this.

### Cerebral Hæmorrhage; Delayed Coma with Rising Blood-pressure.

This case, admitted to the Metropolitan Hospital under my care, presented several obscure features. The patient, a man of 47, suffered from rheumatic fever at the age of 25. There had been alcoholic excess also. Ten days before admission he complained of numbness in the right foot. This passed off, but on the day of admission he suddenly began to drag his right foot. He tried to say that his foot was getting bad again, but in the middle of the sentence he became aphasic. He was found to have right hemiplegia, and his eyes deviated to the left. The left optic disc was rather swollen, and there was an offensive discharge from his right ear. He had incomplete aphasia, and was unable to protrude his tongue. He was evidently able to understand all that was said to him. No disturbance of sensation could be made out. The reflexes were generally exaggerated, except the epigastric, which were absent. There was incontinence of urine and fæces. The heart's apex was one inch outside the nipple line in the sixth space, and the radial artery was hard and tortuous. The blood-pressure was 225 mm., and the urine contained much albumen.

Had he gradually become comatose in the course of a few hours, there would have been little doubt in the mind of anyone that cerebral hæmorrhage had occurred. But he passed into a condition of cerebral irritation; the temperature rose to 101° F., and the pulse quickened to 132. A blood count showed 17,000 leucocytes, rising to 24,000 the next day. The blood-pressure had risen to 245 mm.

The possibility of some septic complication resulting from the aural discharge was discussed. Yet a cerebral abscess from right-sided otitis media could hardly cause a right hemiplegia, nor could sinus thrombosis. Another possibility was an infective endocarditis, set up by a blood infection from the otitis, which had resulted in a septic infarct in the brain. But this would not explain the high and rising blood-pressure, and a blood culture was sterile, while lumbar puncture merely yielded a straw-coloured fluid containing a few lymphocytes. The sole remaining alternative was that the temperature and the leucocytosis were caused by the otitis, and had no connection with the intracranial lesion. There is a natural aversion to diagnosing two separate conditions if a single one will account for all the symptoms, yet in this case the conclusion was justified by events.

Four days later coma gradually ensued, but so slow was its development that the patient could still understand and try to answer questions nine days after the onset of the hemiplegia. Still more curious was the fact that as he became more comatose the blood-pressure fell, the readings on successive days being 175, 155, 130, 125. The temperature, after reaching 102.4°, fell and was normal for the last week of life. He ultimately died 15 days after the onset of the hemiplegia, being completely comatose for the last four or five days of life.

At the post-mortem the vessels at the base of the brain were found to be slightly atheromatous in places; there was no sign of embolism or thrombosis. No softening or discoloration of the brain was to be seen externally. On section a large hæmatoma, composed of concentrically laminated clot, occupied the left basal ganglia. Only a little of the head of the caudate nucleus remained, the lenticular nucleus, optic thalamus, and adjoining white matter including the whole of the internal capsule, being completely destroyed. The brain substance for about half an inch around the hæmatoma was red and soft. A little discoloured fluid occupied the lateral ventricles, but no large hæmorrhage had taken place into them. The right ear contained some thick yellow pus, and the bone was carious.

The heart weighed 19 ounces; the left ventricle was hypertrophied, the mitral valve was thickened, its cusps adherent, and the orifice quite narrow. A row of small firm vegetations studded the auricular aspect of the valve. The kidneys were typically granular; the heart's blood was sterile.

Had the condition of the mitral valve given rise to a murmur during life, the diagnosis of a septic embolus certainly would have been made. Yet it would have been wrong. The blood must have been extravasated so slowly that clotting barred its passage to the lateral ventricles. In this way coma was delayed until there was a large mass of blood in the basal ganglia, but the increased intracranial pressure still produced its ordinary effect of evoking a rise of general blood-pressure. The final fall of pressure with deepening coma must have been due to the cessation of hæmorrhage and the spread of the softening around.

### The Value of Blood-pressure Estimations.

The mechanism by which a rise of blood-pressure inevitably follows a cerebral hæmorrhage is clearly demonstrated by Cushing's experiments. Medullary anæmia would mean death, so that the blood-pressure must be kept above the intracranial to prevent this. The slightest degree of anæmia in the medulla throws the vaso-motor centre into increased activity. Cushing raised the intracranial pressure in animals by introducing normal saline solution into the cranial cavity from a pressure-bottle. Until the intracranial pressure reached the level of the blood-pressure very little effect was produced, but at that point the blood-pressure was at once raised until it was again greater than the intracranial. This was repeated with each increase of intracranial pressure until the vaso-motor centre began to show signs of giving way. The absolute necessity of maintaining the blood-pressure at a higher level than the intracranial establishes a vicious circle, for the hæmorrhage produces a rise of pressure and the rise of pressure increases the hæmorrhage. To lower blood-pressure with the view of checking hæmorrhage is to run the risk of producing medullary anæmia, with possibly fatal results, as Leonard Williams has urged. Is there any way of lowering intracranial pressure directly? Then the vaso-motor centre would allow the blood-pressure to fall; this would assist the arrest of hæmorrhage, and the vicious circle would be broken. Lumbar puncture will diminish intracranial tension and has, therefore, been recommended in such cases. It has been thought to be risky, in that it will leave the arteries less supported, and therefore more liable to bleed. My answer to this is that as soon as the intracranial pressure is reduced the blood-pressure will fall, and therefore the liability to hæmorrhage is diminished. That the blood-pressure can be reduced in this way I have had an opportunity of observing recently. A man in whom I had diagnosed cerebral hæmorrhage had a blood-pressure rising from 165 to 210mm. Lumbar puncture withdrew blood-stained cerebro-spinal fluid. The pressure fell at once to 175 and then more gradually to 135mm., while consciousness was soon regained.

### Cerebral Thrombosis.

A man aged 64 was admitted under my care with the history that for three months he had suffered from headache; he had become very irritable and easily worried. For a fortnight he had vomited each time he had taken food; for the last two days he had been drowsy and rambling in his speech. There was nothing of importance in his past history except an attack of syphilis at 15. His face was expressionless and he was roused with difficulty. There was no optic neuritis, but some old choroiditis. The ear-drums were natural. The heart was slow and feeble, the pulse small; the blood-pressure was 130mm.; the urine was free from albumen. No definite paralysis could be made out, and the reflexes appeared to be normal. Lumbar puncture yielded a clear fluid containing 40 lymphocytes per cubic mm. In view of the long prodromal symptoms, the feeble circulation, and the absence of increased pressure, the diagnosis of cerebral thrombosis was made. His condition did not improve; there was incontinence of urine and fæces, the drowsiness increased, and the facial muscles became very weak. The blood-pressure remained at 130.

The orthodox treatment in senile cerebral thrombosis is to employ cardiac stimulants. But Grainger Stewart has shown that the fatal issue is often due to a rise of pressure in the stage of reaction, or as a result of this stimulant treatment, which bursts the now softened vessel. Bearing this danger in mind, I have been loath to stimulate in cases of cerebral thrombosis. Recently, however, I have heard this method extolled by a competent authority, and as I felt sure that this patient would die if left alone, I thought that it would be better to run the risk. He was accordingly put on a mixture of nitrous ether, caffein, and strychnine every six hours, and two ounces of brandy in the day. He did not improve, however, and a week after admission he became very cyanosed; the pulse is recorded as "quite full"; his temperature rose to 102.6, and he died. I did not see him in this stage, and unfortunately no observation of the blood-pressure was made after the pulse changed in character.

The post-mortem revealed an old clot in the torcula herophili, which had spread more recently into the left lateral sinus. There was a large flat hæmatoma over the left cerebral hemisphere; the blood was of a rusty red colour with partly-decolourised clots; internally it was covered by a thin membrane, the dura forming the outer covering. The cyst did not cross the middle line. The superior longitudinal sinus was patent. The left half of the cerebrum showed a well-marked depression corresponding to the cyst. There were a number of small recent hæmorrhages into the pons and crura cerebri. The heart was slightly enlarged and very flabby; the aortic valves were rather atheromatous. The kidneys contained several small cysts and their surface was slightly granular.

This was evidently an example of the so-called hæmatoma of the dura mater, first described by Virchow. It is usually associated with atrophy of the convolutions, as in this case. As might be expected, therefore, it is more common in asylum than in ordinary practice. The failure of the blood-pressure to rise strongly suggests that the formation of the cyst is compensatory to the atrophy of the convolutions, otherwise it must increase the intracranial pressure, which would raise the blood-pressure. I believe that the change in the character of the pulse and the rise of temperature corresponded to the pontine and crural hæmorrhages, and I cannot but suspect that the stimulant treatment must have been at least in part responsible for their occurrence. At any rate the case has impressed on my mind the reality of the dangers pointed out by Grainger Stewart.

In conclusion, I must express my thanks to those who have kindly allowed me to illustrate these remarks from cases under their care.

